# Association of Low Vitamin D and Intact Parathyroid Hormone (iPTH) in Nepalese Population: When Does iPTH Exactly Rise?

**DOI:** 10.1210/jendso/bvad143

**Published:** 2024-02-20

**Authors:** Sujata Baidya, Eans Tara Tuladhar, Vijay Kumar Sharma, Raju Kumar Dubey, Mithileshwer Raut, Aseem Bhattarai, Naresh Parajuli, Apeksha Niraula

**Affiliations:** Department of Clinical Biochemistry, Institute of Medicine, Maharajgunj Medical Campus, Tribhuvan University, Kathmandu 44600, Nepal; Department of Clinical Biochemistry, Institute of Medicine, Maharajgunj Medical Campus, Tribhuvan University, Kathmandu 44600, Nepal; Department of Clinical Biochemistry, Institute of Medicine, Maharajgunj Medical Campus, Tribhuvan University, Kathmandu 44600, Nepal; Department of Clinical Biochemistry, Institute of Medicine, Maharajgunj Medical Campus, Tribhuvan University, Kathmandu 44600, Nepal; Department of Clinical Biochemistry, Institute of Medicine, Maharajgunj Medical Campus, Tribhuvan University, Kathmandu 44600, Nepal; Department of Clinical Biochemistry, Institute of Medicine, Maharajgunj Medical Campus, Tribhuvan University, Kathmandu 44600, Nepal; Department of Internal Medicine (Endocrinology), Institute of Medicine, Tribhuvan University Teaching Hospital, Kathmandu 44600, Nepal; Department of Clinical Biochemistry, Institute of Medicine, Maharajgunj Medical Campus, Tribhuvan University, Kathmandu 44600, Nepal

**Keywords:** intact parathyroid hormone, iPTH, calcium, phosphorus, vitamin D insufficiency, inflection point, bone demineralization

## Abstract

Vitamin D deficiency is a global public health concern that provokes bone demineralization and weakening. In response to the decreased vitamin D level, calcium stores wear out. The homeostatic effect of compensatory hyperparathyroidism in vitamin D deficiency incites variable discrepancies in different populations. This study intends to decipher the transition point of PTH in relation to levels of vitamin D in a Nepalese population. A cross-sectional study was carried out at Tribhuvan University Teaching Hospital, Nepal. Serum calcium, phosphorus, intact PTH, and 25-hydroxy vitamin D levels were assayed in an Abbott ARCHITECT Integrated System. A correlation plot of PTH and vitamin D was analyzed in Statistical Package for Social Sciences version 22.0. Using a locally weighted scatter plot smoothing method, the relation between these variables was presented graphically. Among 281 individuals, 30.2% had vitamin D levels below 20 ng/mL. There was an archetypical transition in the PTH levels in concert with the decrease in vitamin D level marked by 2 inflection points (ie, 18.5 and 42.0 ng/mL). Our findings suggest that to augment overall health and avert weakness due to vitamin D deficiency, the levels of vitamin D should be maintained above 42.0 ng/mL in our population.

Calcium and phosphorus homeostasis is important in the maintenance of musculoskeletal well-being. This also involves the maintenance of the body's overall homeostasis and refinement of the neurological and cardiovascular system [1]. A symphonious regulation of PTH, vitamin D, and fibroblast growth factor-23 maintains calcium and phosphorus homeostasis [2-4]. The status of 25-hydroxy vitamin D, the major circulating form in the body, varies with geographical location, latitude, sex, race, ethnicity, genetic predisposition/variations, and many other factors [5-8]. Vitamin D inadequacy manifests several communicable and noncommunicable diseases like rickets, colon cancer, breast cancer, diabetes mellitus, autoimmune diseases, tuberculosis, Parkinson's disease, Alzheimer's disease, and others [9, 10]. In response to reduced calcium and vitamin D levels in concert, calcium-sensing receptors in the parathyroid cells upregulate the secretion of PTH [11]. Continuous release of PTH evokes bone demineralization in order to compensate for hypocalcemia [12]. Similarly, the levels of PTH equate the status of vitamin D levels in the body [13]. There is no consensus regarding optimum serum vitamin D concentration or definition of vitamin D deficiency and vitamin D insufficiency [14]. According to the Institute of Medicine (US), a vitamin D level below 30 nmol/L (12 ng/mL) is termed vitamin D deficiency and a level between 30 and 50 nmol/L (12–20 ng/mL) is termed vitamin D insufficiency [14]. There is no well-defined cutoff with regard to the Nepalese population. In Nepalese rural women, 6.3% were found to have vitamin D deficiency while 42.4% had insufficiency following this definition [15]. On the other hand, with the threshold of 20 ng/mL, 55.9% of the population in eastern Nepal had vitamin D deficiency [16]. There is a paucity of data in stating clinical decision levels for vitamin D in individual age groups and across various ethnicities [17, 18]. This study was an attempt to analyze the point of inflection of intact PTH (iPTH) to speculate on critical levels of vitamin D insufficiency in a Nepalese population.

## Materials and Methods

### Study Population

This was a hospital-based cross-sectional study conducted between January 2022 and June 2022 in Tribhuvan University Teaching Hospital, Kathmandu (27.72° N, 85.32° E), Nepal. Patients on a routine visit whose laboratory investigations were sent to the clinical biochemistry laboratory were recruited. The subjects included in the study were not under any calcium or vitamin D supplementation. Pregnant women and individuals on any medications altering bone mineral metabolism were excluded.

### Sample Size

The sample size was calculated using the formula $\mathrm{SS}=\frac{z^{2}(p)(1-p)}{D^{2}}$

where z = standard normal prevalence at 95% confidence interval = 1.96, p = prevalence rate = 69.6% = 0.696 [19], D = type I error = 5% = 0.05. A total of 310 samples was included in the study.

### Definition and Criteria

The reference range for different parameters used were calcium (2.1-2.6 mmol/L), phosphorus (2.5-4.8 mg/dL), iPTH (15-68 pg/mL). A level of 25-hydroxy vitamin D less than 20 ng/mL was considered deficient, 20-29 ng/mL insufficient, above 30 ng/mL sufficient, and values exceeding 150 ng/mL intoxication [1].

### Analysis of Laboratory Parameters

Serum calcium and phosphorus were measured by spectrophotometric method and serum iPTH and 25-hydroxy (25-OH) vitamin D by chemiluminescence microparticle immunoassay in the Abbott ARCHITECT Integrated System.

### Statistical Analyses

The data was entered in Microsoft Excel and analyzed using Statistical Package for Social Sciences (version 22.0). Descriptive statistics were presented as mean ± SD, median, and interquartile range. Correlation of the study variables was assessed using Spearmann's correlation coefficient. The relation between PTH and 25-OH vitamin D was presented graphically using the locally weighted scatter plot smoothing (LOWESS) curve method. Multiple regression analysis was done to find the association of age, calcium, phosphorus, and vitamin D with PTH. A *P*-value of less than .05 was considered statistically significant.

### Ethical Issues and Considerations

This was a laboratory-based noninterventional study, and, as per rules and regulation of the organization, ethical clearance for utilization of clinical data was granted from the Institutional Review Committee of the Institute of Medicine (Reference No. 322 [6-11] E^2^ 078/079).

## Results

A total of 281 patients were included in the study, among which 85 individuals (30.2%) had 25-OH vitamin D levels below 20 ng/mL and 196 individuals had values above 20 ng/mL. Out of the total enrolled participants, males were preponderant (57.3%, n = 161). There were 55 individuals with high serum creatinine.

The median PTH values of the individuals with 25-OH vitamin D < 20 ng/mL was higher as compared to those with levels above 20 ng/mL among the individuals with normal as well as high creatinine. The median calcium level of those with vitamin D less than 20 ng/mL was lower than those with vitamin D higher than 20 ng/mL (Table 1). However, it was of note that individuals with high creatinine levels had comparatively higher median PTH values as well as phosphorus levels though statistically insignificant.

**Table 1.** Values of PTH, calcium, and phosphorus at different levels of 25-hydroxy vitamin D

Creatinine (µmol/L) (A)	Vitamin D level (ng/mL) (B)	PTH (pg/mL) (C)	Calcium (mmol/L) (D)	Phosphorus (mg/dL) (E)
≤110	<20 (n = 71)	224.1 (111.7, 353.7)	1.78 ± 0.24	5.7 (4.4, 7.2)
	20–30 (n = 60)	127.9 (53.8, 340.3)	1.9 ± 0.33	4.8 (3.6, 6.1)
	30–100 (n = 92)	111.0 (42.4, 300.3)	1.9 ± 0.28	4.5 (3.6, 6.1)
	>100 (n = 3)	35.8 (15.5, 9.37)	2.2 ± 0.10	3.4 (3.2, 5.2)
>110	<20 (n = 14)	201.9 (97.8, 421.8)	1.78 ± 0.28	5.6 (4.6, 6.2)
	20–30 (n = 16)	189.7 (113.5, 316.7)	1.9 ± 0.24	4.3 (3.3, 5.4)
	30–100 (n = 24)	137.1 (54.3, 354.4)	1.9 ± 0.25	5.2 (3.9, 5.9)
	>100 (n = 1)	744.7	2.0	5.5

A vs B (Chi-square test) = 0.851.

A vs C (Mann–Whitney U test) = 0.333.

A vs D (Student's t-test) = 0.164.

A vs E (Mann–Whitney U test) = 0.590.

The study participants comprised a wide range of age groups ranging from children less than 5 years to a maximum of 83 years with the bulk of participants from the adult age group. The median value of PTH in adolescent males was comparatively higher as compared to other age groups. However, the vitamin D levels were comparable in all age groups as shown in Table 2.

**Table 2.** Distribution of vitamin D and PTH according to age and sex

Age group	Vitamin D		PTH	
	Male	Female	Male	Female
Children (<13 years) (n = 9)	29.95 (20.12, 44.4)	12.3	72.8 (22.6, 195.18)	1604
Adolescents (13-25 years) (n = 40)	24 (17.0, 38.3)	27.3 (21.45, 38.25)	242.6 (74.1, 440.7)	196.4 (48.85, 875.85)
Adults (25-60 years) (n = 151)	26.2 (18.4, 48.6)	26.0 (17.8, 37.5)	220 (56.7, 375.2)	124.1 (54.1, 381.15)
Elderly (>60 years) (n = 80)	25.4 (19.2, 40.9)	29.9 (19.4, 37.7)	171.0 (78.6, 297.3)	161.0 (69.9, 337.3)

**Figure 1.**
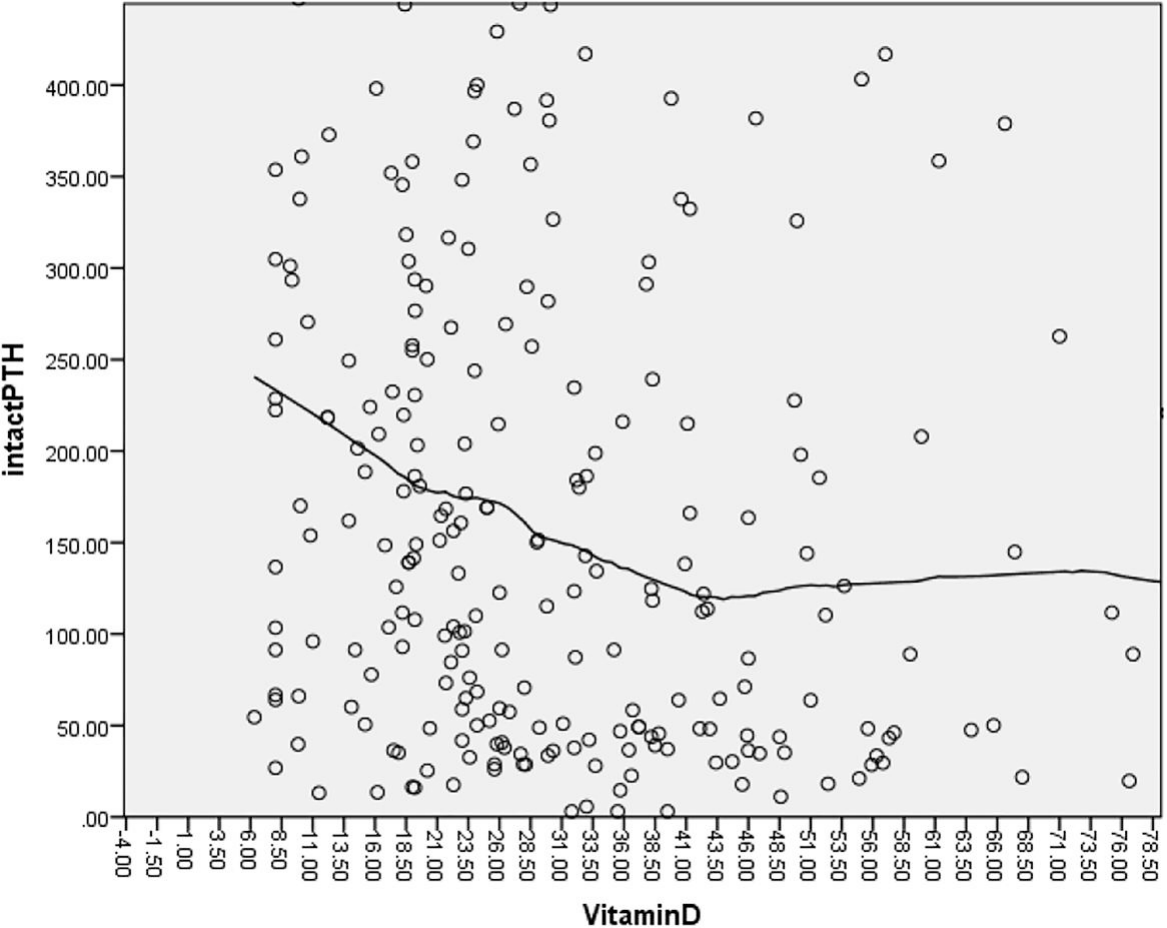
Correlation between PTH and vitamin D (LOWESS method).

There was a negative correlation between PTH and vitamin D using Spearmann's correlation (r = −0.224, *P*-value <.001). The data on scatter plots of PTH and vitamin D was fit using the LOWESS method to generate a smooth curve (Fig. 1). By analyzing the LOWESS curve visually, 2 inflection points of paradigm shift in PTH were observed. Below a vitamin D level of 42.0  ng/mL, PTH levels began to rise slowly, while there is a plateau formed beyond this level. On the other hand, as the vitamin D level decreased to 18.5 ng/mL, there was a steep rise in PTH levels. Multiple regression analysis showed that only calcium was significantly associated with PTH levels (Table 3).

**Table 3.** Multiple linear regression of study variables

Variables	Coefficient	SE	Standardized B	t value	*P*-value
Intercept	832.61	129.73		6.418	<.001^a^
Age	−1.53	0.90	−0.109	−1.695	.091
Calcium	−266.78	61.57	−0.286	−4.333	<.001^a^
Phosphorus	5.10	3.07	0.105	1.663	.098
Vitamin D	−1.492	0.849	−0.115	−1.757	.080

^a^ *P*-value significant below .05.

## Discussion

In our study, a total of 85 individuals among 281 had a 25-OH vitamin D level below 20 ng/mL. There is a very high prevalence of vitamin D deficiency in the Nepalese population ranging from 32.0% to 73.6% with female preponderance [16, 20-22]. However, the definition of deficiency varies in different studies. We report the prevalence of vitamin D deficiency in 30.2%, which is close enough to the findings of Chandra et al [20], which defines vitamin D deficiency when vitamin D levels are below 15 ng/mL. Other studies state below 20 ng/mL [16, 21] and 30 ng/mL [22] as deficiencies of vitamin D.

Vitamin D deficiency incites secondary hyperparathyroidism, which cascades a series of events leading to bone loss and deformities [17]. There was a nonlinear association between 25-OH vitamin D and PTH in our study. Our findings speculate a shift in PTH levels at 2 different levels of vitamin D (ie, 18.5 ng/mL and 42.0 ng/mL). There was a steeper rise in PTH level when the vitamin D level fell below 18.5 ng/mL, while a plateau formed after a vitamin D rise above 42.0 ng/mL.

These values marginally corroborate with the study by Mukhopadhyay et al done in a similar latitude (26.6687°N, 87.6828°E) in India with a less conspicuous rise in a vitamin D level of 32.0 ng/mL and a steep rise below 16.5 ng/mL [23]. However, the level of vitamin D in our study at which PTH reaches a plateau is a bit higher compared to that study. Despite the similarity in latitude, the disparity in the point at which PTH normalizes may be due to a different study population, which included a rural tribal population in the other study, while our study used an urban population that is presumably confined to staying indoors.

There was a negative correlation between PTH and vitamin D in our study that aligns with an evident inverse relationship of these 2 variables until vitamin D reaches 30 to 40 ng/mL [24]. Similar to our findings, a retrospective data mining review stated that maximal suppression of PTH in children was found to be at a vitamin D level of 30 ng/mL [18]. In contrast to our study, a study done in South Korea by Kang et al demonstrated that the vitamin D level for maximal suppression of PTH in children and adolescents was 18 ng/mL [25]. Despite being far from the equatorial line compared to Nepal, the lower vitamin D levels for suppression of PTH in South Koreans may be due to lifestyles, body mass index, and genetic and biosocial variation accustomed to exposure to sunlight [26, 27]. A similar finding of genetic predisposition was theorized in a study by Dawodu et al in which, despite participants inhabiting the same environment, levels of vitamin D were lower in the overall population in the United Arab Emirates compared to European contingents [28]. Other than the variation in geography, the discrepancies in results might be seen due to different detection methods used in various studies like electrochemiluminesence [25, 27], immunoradiometric, and chemiluminescence assay [26, 28].

There is a dilemma in whether serum PTH suppression can be an appropriate measure to assess the optimal vitamin D status in children and adolescents because, mostly in adults, higher PTH is often associated with bone resorption. On the other hand, there is no well-established reference range for PTH in children and adolescents [26]. This study, however, does not clarify the inflection of PTH and vitamin D in different stages of life, which might also alter the threshold of clinical decision levels. There are some other limitations in our study in terms of exploring other determinants or etiologies of vitamin D deficiency including diet, physical activity, seasonal variation, and factors like body mass index and magnesium levels. This study paves the way for further studies in the Nepali population regarding the recommended dose and duration for vitamin D and calcium supplementation for optimum PTH levels. It is recommended to analyze the threshold level of PTH suppression more meticulously in special populations like postmenopausal, pregnant, and lactating women and diverse populations across geographical belts and ethnic groups in parallel with radiological findings.

There is a slow rise in iPTH just as the vitamin D level falls below 42.0 ng/mL, and it shoots up briskly as it reaches 18.5 ng/mL. In order to avert weakness as well as immune deficiency and augment overall health, levels of vitamin D should be maintained above 42.0 ng/mL, while levels below 18.5 ng/mL may be termed deficiency in the Nepalese population.

## Data Availability

Some or all datasets generated during and/or analyzed during the current study are not publicly available but are available from the corresponding author on reasonable request.
